# Intoxication with GHB/GBL: characteristics and trends from ambulance-attended overdoses

**DOI:** 10.1186/s13049-017-0441-6

**Published:** 2017-09-22

**Authors:** Desiree Madah-Amiri, Lars Myrmel, Guttorm Brattebø

**Affiliations:** 1 The Norwegian Centre for Addiction Research, The University of Oslo, Oslo, Norway; 2 Bergen Emergency Medical Services, Department of Anesthesia and Intensive Care, Haukeland University Hospital, Bergen, Norway; 3 Department of Clinical Medicine, University of Bergen, Bergen, Norway

**Keywords:** GHB, GBL, Party drugs, Club drugs, Intoxication, Overdoses, Prehospital care, Poisoning, Ambulance

## Abstract

**Background:**

Overdoses from so-called “club drugs” (GHB/GBL) have become a more frequent cause of overdoses attended by ambulance services. Given its availability, affordability, and lack of awareness of risks, there is a common misconception among users that the drug is relatively safe.

**Methods:**

This study reviewed ambulance records in Bergen, Norway between 2009 and 2015 for cases of acute poisonings, particularly from suspected GHB/GBL intoxication.

**Results:**

In total, 1112 cases of GHB and GBL poisoning were identified. GHB was suspected for 995 (89%) of the patients. Men made up the majority of the cases (*n* = 752, 67.6%) with a median age of 27 years old. Temporal trends for GHB/GBL overdoses displayed a late-night, weekend pattern. The most frequent initial symptoms reported were unconsciousness, or reduced consciousness. Most of the patients required further treatment and transport. During the period from 2009 to 2015, there was a nearly 50% decrease in GHB/GBL overdoses from 2013 to 2014.

**Discussion:**

The characteristics of GHB/GBL overdose victims shed light on this patient group. The decrease in incidence over the years may be partly due to a legal ban on GBL in Norway, declared in 2010. It may also be due to an increase in the use of MDMA/ecstasy.

**Conclusion:**

The review of ambulance records on the prehospital treatment of overdoses can be beneficial in monitoring, preparing, and prevention efforts aimed to benefit this vulnerable group.

## Background

Intoxication is a frequent cause of calls to emergency medical services (EMS) in Norway, with most of these poisonings being self-inflicted [1–3]. Primarily these cases involve alcohol and opioids, particularly heroin [4, 5]. However, in recent years, Scandinavia has experienced overdoses due to so-called “club drugs” (gamma-hydroxybuturate (GHB) and gamma-butyrolactone (GBL)) [6–9].

Gamma-hydroxybutyrate is found naturally occurring in many tissues of the body, and is closely related to the inhibitory neurotransmitter gamma-aminobutyric acid (GABA) [10]. The sodium salt of gamma-hydroxybutyric acid is called gamma-hydroxybuturate (GHB). It is likely that GHB has its own GHB receptor, and when in high doses works to activate GABA B receptors, including dopamine release in the brain [11, 12]. GABA-B receptors are likely activated both in the pre- and post-synapses, which may explain many of the different effects of GHB intoxication [13].

The drug was originally developed in the 1960s in an attempt to create a GABA analog that would cross the blood-brain barrier [11]. When the compound proved to have strong sedative properties, it was then considered for its use as an anesthetic agent. Given the narrow therapeutic interval, combined with large individual differences in tolerance, today the drug has a very limited medicinal use [14, 15]. The calming and euphoric effects of GHB in low doses (20–30 mg/kg) have given the drug the nickname “liquid ecstasy” [16]. Since the drug is both inexpensive and easy to ingest, it has been popular as a “party drug.”

The oral absorption of GHB is relatively fast, with peak concentrations in plasma after 20–45 min, and half-life of 20–30 min [17]. At higher doses (> 50 mg/kg), the hypnotic effects are more prominent, and at doses >60 mg/kg coma, convulsions, and respiratory depression can occur [16]. The clinical hallmark of GHB poisoning is rapid onset of coma, with respiratory depression, hypoventilation and bradycardia [11–13]. Combination with alcohol potentiates these effects, especially respiratory depression and hypotension [10, 18].

GHB is a metabolic precursor to gamma-butyrolactone (GBL) and 1.4-butanediol (1.4 BD). GBL is used in the chemical industry, and is relatively easily acquired via the internet. Therefore, it has been imported to Norway in large quantities, predominately from Eastern Europe (personal communication, Hordaland Police District). The drug may be either “cooked” into GHB by the addition of caustic soda, or ingested directly, since after oral ingestion the substance is rapidly converted to GHB [19]. GBL appears to be more potent than GHB, as animal studies have shown that the substance at equimolar oral doses gave both a faster and longer-lasting effect than GHB, as well as a higher peak plasma concentration [20, 21]. Increasing occurrences of fatal poisonings and deaths in which GBL was probably involved, as well as more international focus on this drug was the reason that also GBL was classified as a narcotic and therefore prohibited in Norway in March 2010 [22].

The intoxicating effects of GHB are well known, and it has been reported to be involved in varying rates of fatal poisonings [8, 23, 24]. In Norway, opioids are most commonly taken parenterally and are significantly more costly, lending to a lower threshold to try the innocent appearing “soda caps” with GHB. GHB is often used recreationally as a party drug, with typical users viewing the drug as relatively non-toxic and harmless, comparable to alcohol [25].

Ambulance records have been used to investigate epidemiological trends for a variety of issues [26–28]. Population level monitoring of GHB use is limited [29], and the indirect information provided by ambulance records can provide a current overview of GHB overdoses in the city. This information can give insight into distinct user groups and patterns of use, which can in turn be used to monitor and guide treatment and prevention interventions. Further, an investigation into temporal patterns may shed light on what role the GBL restriction had on overdoses. Therefore, the aims of this study were to investigate characteristics and temporal trends of GHB/GBL ambulance attended overdoses in Bergen from 2009 to 2015.

## Methods

### Setting and design

This retrospective study reviewed emergency dispatch records and ambulance records from the Bergen Emergency Medical Services (EMS) from 2009 to 2015. All patients treated by Bergen EMS during this period for a suspected opioid or GHB/GBL overdose were included in the study.

Bergen is the second largest city in Norway, with a population of approximately 275,000 [30]. Although alcohol and opioid overdoses make up the majority of the calls for poisonings, in 2008 ambulances began to notice an increase in overdoses with GHB/GBL as the presumed cause.

### Data source

Emergency calls are coded based on caller information and entered electronically into a database. These call codes do not sufficiently capture all overdose events, so a manual review through all ambulance dispatch call entries was completed. Patients that responded to naloxone, an opioid antagonist, were considered to have had an opioid overdose and were therefore not included in this study. Overdoses with GHB/GBL were based on information from the caller and the EMS staff suspicion for GHB/GBL intoxications (including the presence of “paraphernalia” at the scene). All data files were anonymous, and stored on a secured data server. No linkage to hospital patient’s records or other data registries was performed.

### Measures

The ambulance staff routinely completes paper-based patient records for all patient encounters. Several key variables about suspected overdoses were extracted from the caller-database and patient records. These included: date, time, age and gender of the patient, location, the presenting symptoms, suspected drug ingested, and to where the patient was transported if not treated and left at scene.

### Statistical analysis

Frequency analyses were used to describe many of the outcome measures. Chi-square tests were used to compare frequencies, and Fisher’s exact test was reported if cell frequencies were less than five. Medians were reported when the data was not normally distributed. Age comparison with gender was done using the Mann-Whitney U test. A *p*-value of <0.05 was considered significant. Statistical analyses were preformed using SPSS software version 22.

## Results

### Demographics

During the study period, a total of 1112 poisoning call-outs for GHB or GBL overdoses. Males made up 67.6% (Table 1). The median age was 26 (range 13–64). There was one 64-year old patient who had ingested GHB accidentally. Disregarding this case, the oldest victim with a GHB/GBL overdose was 62 years. The majority of the patients reported taking only GHB (*n* = 883, 79.4%), with 130 (11.7%) reporting to have taken GHB or GBL along with another substance (benzodiazepines, alcohol, amphetamines, or alcohol). There were 99 cases (8.9%) with that suspected only GBL. Males were significantly older than females for the GHB patients (*p* < 0.001). There was no significant age difference among the genders for the GBL patients (*p* = 0.50).

**Table 1:** Characteristics of GHB and GBL overdose patients attended by Bergen ambulance services from 2009 to 2015

	GHB/GBL overdose patient
Gender	N (%)
Male	752 (67.6)
Female	311 (28.2)
Missing	49 (4.4)
Total	1112 (100)
Age	Median (IQR)
Male	27 (23–33)
Female	25 (21–230)

*IQR* inter quartile range

### Reported symptoms

Reported symptoms are listed in Table 2. This shows the symptoms of the GHB/GBL overdose, based on the patient records completed by the ambulance service. The majority of patients were unconscious or had reduced consciousness (*n* = 944, 84.9%) when ambulance staff examined them. There were 103 cases (9.3%) that presented with irritated or agitated behavior, somewhat characteristic of a GHB overdose.

**Table 2:** Symptoms of a GHB/GBL overdose based on ambulance patient records

Symptom	N (%)
Unconscious (GCS 3–7)	662 (59.5)
Reduced consciousness	282 (25.4)
Irritated	103 (9.3)
Respiratory issues	24 (2.2)
Cardiac issues	9 (0.8)
Other	31 (2.8)
Deceased	1 (0.1)

*GCS* Glasgow Coma Scale

### Temporal characteristics

**Fig. 1:**
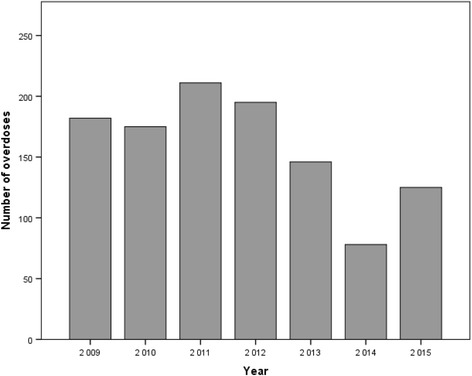
Annual GHB/GBL overdoses attended by Bergen ambulance services from 2009 to 2015

Overdoses in 2009–2013 were relatively stable, with a peak in 2011 (*n* = 211, 19%) (Fig. 1). In 2014 a sharp decrease was seen. From 2013 (*n* = 146, 13.1) to 2014 (*n* = 78, 7.0%) the number of suspected GHB/GBL intoxication cases fell by nearly 50%. There was significant variation among the different days of the week for GHB/GBL overdoses (*p* < 0.001). Mondays had the lowest number of ambulance call-outs (*n* = 96, 8.6%), and weekends had the highest (Saturdays (*n* = 196, 17.6%) and Sundays (*n* = 199, 17.9%)).

**Fig. 2:**
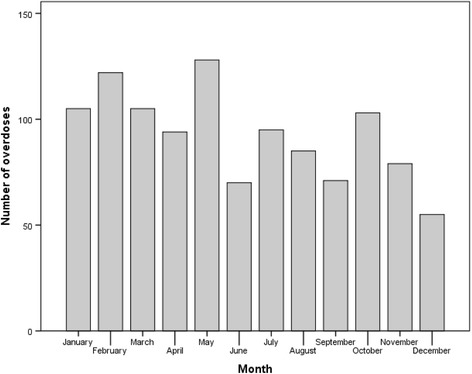
Monthly variation for GHB/GBL overdoses attended by Bergen ambulance services from 2009 to 2015

Over 40% of the overdoses (*n* = 451) occurred between 22:00 and 4:00 in the morning. There was a pronounced peak after midnight, at 1:00 am (*n* = 102, 9.2%). Significant monthly variation was observed (p < 0.001), with a peak in February (*n* = 122, 11.0%) and May (*n* = 128, 11.5%) and the lowest in June (*n* = 70, 6.3%) and December (*n* = 55, 4.9%) (Fig. 2).

### Disposition

Disposition for the patients included transport to the emergency department, hospital, or treated at the scene. Nearly all patients with a suspected GHB/GBL overdose required further transport (*n* = 1041, 93.6%). There were 71 cases (6.4%) that were treated and left at scene. The numbers of deaths are under-reported, as the ambulance services normally are not involved if the patient is dead at the scene. From other sources (police), there are known to be two other deaths due to GHB/GBL overdoses during the period. Outcomes following admission and treatment at the hospital were not investigated.

## Discussion

Fatal poisoning from drug overdoses is frequent in Scandinavia [9, 31], as in many cities. Although alcohol and opioids account for the majority of the substances reported for these events, in recent years GHB/GBL overdoses have also been a significant concern. Through this epidemiological analysis of ambulance trends, characteristics about the GHB/GBL patients and the circumstances surrounding their overdoses were explored. In general, these patients were male, in their mid-20s, and found unconscious at the scene. The temporal patterns suggest party use, being most frequent during late-night and weekends.

The characteristics and incidences of these GHB/GBL overdoses, when compared with opioid overdoses show notable differences. The GHB/GBL victims are younger than opioid overdose victims, and are hospitalized more often [32]. This age difference is consistent with what others have found, demonstrating a younger demographic [1, 7, 23, 33]. Gender distribution was similar to opioid overdoses [32, 34], with an approximate 70% to 30% male to female ratio. Further, seasonal differences between opioid overdoses and GHB/GBL overdoses were observed. Others have reported a summer peak for opioid overdoses [32], however this study found June to be the month with the lowest incidence of GHB/GBL overdoses.

The reported symptoms for a GHB/GBL overdose correspond to findings from other studies [13]. Unconsciousness and reduced consciousness were the most common symptoms, with over 85% of patients being in this group. A small portion (9%) was registered as irritated or agitated, which also corresponds with the findings in other studies [35]. The proportion is probably lower than for other drug-related/poisoning events (for example, with alcohol), since many patients are partially or completely unconscious. Other studies have found alcohol intoxications to include more traumatic injuries [36] and sexual assault [37] than the GHB/GBL patients in this study.

Ambulance attendances for alcohol intoxication showed temporal similarities with GHB/GBL patients. We found a peak in GHB/GBL overdoses during the weekends, similar to alcohol-related road accidents [38] and hospital admissions [39].

Following the ban on GBL in 2010, an eventual decrease in GHB/GBL overdoses was observed beginning in 2012 and continuing through 2015. The incidence shift from 2009 to 2015 shows a significant decrease in GHB/GBL overdoses. In Norway and throughout Europe, increases in the use of ecstasy/MDMA has been observed [31]. While co-consumption of ecstasy and GHB is low [40], polydrug use remains a concern [41]. “Club drug” recreational users have similar user groups, and it is possible that this decreasing GHB trend in Bergen has been influenced by the increasing use of ecstasy/MDMA.

### Limitations

The first limitation in this study is that our results are based on presumed cause of overdose poisoning and there are no confirmatory analyzes of agents. Hence, intoxication due to other drugs mimicking GBH/GBL could have been included. Second, the information from the call center is based on the individual operators’ manual entries. Although this varies, there is little reason to suspect systematic errors in registration. In attempts to capture all cases, emergency call-outs by ambulance during the period were also reviewed manually so that coding errors should not result in underestimation of the presence of serious overdose cases. Third, given the nature of a retrospective analysis, data was missing for some of the variables examined, particularly age of the victim. However, it is little reason to assume that the age of the younger patients would be less available than for older. Despite these limitations, these numbers likely capture a conservative estimate of GHB/GBL occurrences in the area.

## Conclusion

Acute intoxication due to substance use accounts for a significant portion of all acute ambulance call-outs in Bergen during week-ends. Characteristics of overdoses from opioids or GHB/GBL varied in several ways, shedding light on the apparent differences in these distinct patient groups. A focus on this distinct user group can help to guide prevention and outreach efforts, as many may not regularly access low-threshold services. The reduction in GHB/GBL overdoses following the ban on GBL may suggest that prohibition on the use of the drug may have had an effect on the number of overdoses, although this may also be explained by an increase in ecstasy/MDMA use. The review of ambulance records on the prehospital treatment of overdoses can be beneficial in monitoring, preparing, and prevention efforts aimed to benefit this distinct group.

## Abbreviations

EMS: Emergency Medical Services
GABA: Gamma-aminobutyric acid
GBL: Gamma-butyrolactone
GCS: Glasgow Coma Scale
GHB: Gamma-hydroxybuturate
IQR: Inter Quartile Range
MDMA: 3,4-methylenedioxymethamphetamine

## References

[1] Hovda KE (2008). Acute poisonings treated in hospitals in Oslo: a one-year prospective study (I): pattern of poisoning. Clin Toxicol.

[2] Rygnestad T, Fagerhaug O (2004). Acute deliberate self-poisonings in the area of Trondheim, 1978-2002. Tidsskr Nor Laegeforen.

[3] Heyerdahl F (2008). Pre-hospital treatment of acute poisonings in Oslo. BMC Emerg Med.

[4] Lund C (2012). Outpatient treatment of acute poisonings in Oslo: poisoning pattern, factors associated with hospitalization, and mortality. Scand J Trauma Resusc Emerg Med.

[5] Vallersnes OM (2015). Patients presenting with acute poisoning to an outpatient emergency clinic: a one-year observational study in Oslo, Norway. BMC Emerg Med.

[6] Persson SA (2001). GHB--dangerous, addictive and uncontrollable “party drug”. Lakartidningen.

[7] Knudsen K, Greter J, Verdicchio M (2008). High mortality rates among GHB abusers in Western Sweden. Clin Toxicol (Phila).

[8] Knudsen K (2005). GHB, GBL and butanediol poisonings--a serious problem in Western Sweden. Lakartidningen.

[9] Simonsen KW (2015). Fatal poisoning in drug addicts in the Nordic countries in 2012. Forensic Sci Int.

[10] Snead OC, Gibson KM (2005). Gamma-hydroxybutyric acid. N Engl J Med.

[11] Laborit H (1964). SODIUM 4-HYDROXYBUTYRATE. Int J Neuropharmacol.

[12] Dyer JE, Haller CA (2004). Medical toxicology. g-hydroxybutyrate, g-butyramide olactone, and 1.4-butanediol.

[13] Schep LJ (2012). The clinical toxicology of gamma-hydroxybutyrate, gamma-butyrolactone and 1,4-butanediol. Clin Toxicol (Phila).

[14] Vickers MD (1968). Gamma hydroxybutyric acid. Clinical pharmacology and current status. Proc R Soc Med.

[15] Carter LP (2009). Illicit gamma-hydroxybutyrate (GHB) and pharmaceutical sodium oxybate (Xyrem): differences in characteristics and misuse. Drug Alcohol Depend.

[16] Couper FJ, Marinetti LJ (2002). Gamma-Hydroxybutyrate (GHB) - Effects on human performance and behavior. Forensic Sci Rev.

[17] Lenz D, Rothschild MA, Kroner L (2008). Intoxications due to ingestion of gamma-butyrolactone: organ distribution of gamma-hydroxybutyric acid and gamma-butyrolactone. Ther Drug Monit.

[18] Miotto K (2001). Gamma-hydroxybutyric acid: patterns of use, effects and withdrawal. Am J Addict.

[19] Wood DM (2008). Medical and legal confusion surrounding gamma-hydroxybutyrate (GHB) and its precursors gamma-butyrolactone (GBL) and 1,4-butanediol (1,4BD). QJM.

[20] Lettieri J, Fung HL (1978). Improved pharmacological activity via pro-drug modification: comparative pharmacokinetics of sodium gamma-hydroxybutyrate and gamma-butyrolactone. Res Commun Chem Pathol Pharmacol.

[21] Goodwin AK (2009). Behavioral effects and pharmacokinetics of gamma-hydroxybutyrate (GHB) precursors gamma-butyrolactone (GBL) and 1,4-butanediol (1,4-BD) in baboons. Psychopharmacology.

[22] GBL on the narcotic list, in 20/2010, Norwegian Directorate of Health, 2010.

[23] Knudsen K, Jonsson U, Abrahamsson J (2010). Twenty-three deaths with gamma-hydroxybutyrate overdose in western Sweden between 2000 and 2007. Acta Anaesthesiol Scand.

[24] Wong CG (2004). Gamma-hydroxybutyric acid: neurobiology and toxicology of a recreational drug. Toxicol Rev.

[25] Brennan R, Van Hout MC (2014). Gamma-hydroxybutyrate (GHB): a scoping review of pharmacology, toxicology, motives for use, and user groups. J Psychoactive Drugs.

[26] Krayeva YV (2013). Pre-hospital management and outcome of acute poisonings by ambulances in Yekaterinburg, Russia. Clin Toxicol (Phila).

[27] Simpson PM (2013). Epidemiology of ambulance responses to older people who have fallen in New South Wales, Australia. Australas J Ageing.

[28] Lloyd BK, McElwee PR (2011). Trends over time in characteristics of pharmaceutical drug-related ambulance attendances in Melbourne. Drug Alcohol Rev.

[29] Degenhardt L, Copeland J, Dillon P (2005). Recent trends in the use of “club drugs”: an Australian review. Subst Use Misuse.

[30] Estimated population growth and population 2015 [cited 2016 June 9, 2016]; Available from: https://www.ssb.no/en/befolkning/statistikker/folkemengde/aar-berekna/2015-12-17.

[31] European Monitoring Centre for Drugs and Drug Addiction Lisbon (2015). European Drug Report in Trends and Developments.

[32] Madah-Amiri D (2017). Circumstances surrounding non-fatal opioid overdoses attended by ambulance services. Drug Alcohol Rev.

[33] Kim SY (2007). High-risk behaviors and hospitalizations among gamma hydroxybutyrate (GHB) users. Am J Drug Alcohol Abuse.

[34] Knowlton A (2013). EMS runs for suspected opioid overdose: implications for surveillance and prevention. Prehospital Emergency Care.

[35] Liechti ME (2006). Clinical features of gamma-hydroxybutyrate and gamma-butyrolactone toxicity and concomitant drug and alcohol use. Drug Alcohol Depend.

[36] Holzer BM (2012). Ten-year trends in intoxications and requests for emergency ambulance service. Prehosp Emerg Care.

[37] McLaughlin MP (2010). Alcohol-associated illness and injury and ambulance calls in a midwestern college town: a four-year retrospective analysis. Prehosp Emerg Care.

[38] Foster S (2015). Temporal patterns of alcohol consumption and alcohol-related road accidents in young Swiss men: seasonal, weekday and public holiday effects. Alcohol Alcohol.

[39] O'Farrell A (2004). The burden of alcohol misuse on emergency in-patient hospital admissions among residents from a health board region in Ireland. Addiction.

[40] Lott S, Musshoff F, Madea B (2012). Estimation of gamma-hydroxybutyrate (GHB) co-consumption in serum samples of drivers positive for amphetamine or ecstasy. Forensic Sci Int.

[41] Grov C, Kelly BC, Parsons JT (2009). Polydrug use among club-going young adults recruited through time-space sampling. Subst Use Misuse.

